# The Effects of FABP4 on Cardiovascular Disease in the Aging Population

**DOI:** 10.1007/s11883-024-01196-5

**Published:** 2024-05-03

**Authors:** Ellen M. van der Ark-Vonk, Mike V. Puijk, Gerard Pasterkamp, Sander W. van der Laan

**Affiliations:** Central Diagnostics Laboratory, Division Laboratory, Pharmacy, and Biomedical Genetics, University Medical Center Utrecht, University of Utrecht, Utrecht, The Netherlands

**Keywords:** FABP4, Atherosclerosis, Plaque, eQTL, Cardiovascular disease

## Abstract

**Purpose of Review:**

Fatty acid-binding protein 4 (FABP4) plays a role in lipid metabolism and cardiovascular health. In this paper, we cover FABP4 biology, its implications in atherosclerosis from observational studies, genetic factors affecting FABP4 serum levels, and ongoing drug development to target FABP4 and offer insights into future FABP4 research.

**Recent Findings:**

FABP4 impacts cells through JAK2/STAT2 and c-kit pathways, increasing inflammatory and adhesion-related proteins. In addition, FABP4 induces angiogenesis and vascular smooth muscle cell proliferation and migration. FABP4 is established as a reliable predictive biomarker for cardiovascular disease in specific at-risk groups. Genetic studies robustly link *PPARG* and *FABP4* variants to FABP4 serum levels. Considering the potential effects on atherosclerotic lesion development, drug discovery programs have been initiated in search for potent inhibitors of FABP4.

**Summary:**

Elevated FABP4 levels indicate an increased cardiovascular risk and is causally related to acceleration of atherosclerotic disease, However, clinical trials for FABP4 inhibition are lacking, possibly due to concerns about available compounds’ side effects. Further research on FABP4 genetics and its putative causal role in cardiovascular disease is needed, particularly in aging subgroups.

## Introduction

Aging has since long been considered an important factor in the development and progression of atherosclerosis and subsequent events such as myocardial infarction or ischemic stroke [1]. Atherosclerotic plaque size not only increases but also destabilizes with more inflammatory cells and atheromatous lesions that are observed in elderly patients [2]. Although some view(ed) arterial demise due to atherosclerosis as an ‘inevitable destiny,’ just after the Second World War, there was increasing awareness for the social and medical impact of (fatal) atherosclerosis in an aging population and the recognition that more work in the decades to follow was imperative [1]. The decades-old call to study atherosclerosis led to discoveries in the 70 s and 80 s that the activity of the enzymatic proteins prolyl hydroxylase, lysyl oxidase, and collagenase did not change in chickens fed a cholesterol diet compared to controls, rather their enzymatic activity reduced as the chickens grew older [3]. Age-dependent cellular protein activity is now known to influence cell culturing, and cultured vascular smooth muscle cells (SMCs) show reduced low-density lipoprotein (LDL) degradation in cells derived from older donors, and similarly reduced enzymatic activity may modify SMC proliferation and migration [4, 5].

Thus, our vascular proteome changes as we age. Here, we discuss one specific protein, which is equally important for meat quality [6], feed efficiency [7], and milk content variability in livestock [8, 9] as it is for lipid metabolism in health and cardiovascular disease (CVD) in humans: fatty acid-binding protein 4 (FABP4). In this review, we will provide an update on some biology of FABP4, the role of FABP4 in atherosclerotic disease, genetic factors modifying FABP4 levels, drug development, and a future perspective.

## The Protein Family of Fatty Acid-Binding Proteins

FABP4, a 132 amino acid protein, is part of a family of fatty acid-binding proteins (FABPs) consisting of nine isoforms, that play a role in fatty acid solubilization, trafficking, and metabolism in different tissues and organs across species, and are named after the first tissue they were discovered in [10]. Fatty acids are hydrophobic, but soluble in the cytosol when bound inside the β-barrel structure, consisting of 10 anti-parallel β-sheets and capped by helix-turn-helix motif of FABPs. The cytoplasmic chaperones FABPs act as local and systemic mediators in intracellular mechanisms behind metabolism, lipid fluxes, intracellular signaling through interaction with membrane and intracellular proteins (for example, peroxisome proliferator-activated receptors (PPARs) and hormone-sensitive lipase (HSL)), and inflammatory processes [11, 12•, 13].

## FABP4 Biology

**Fig. 1:**
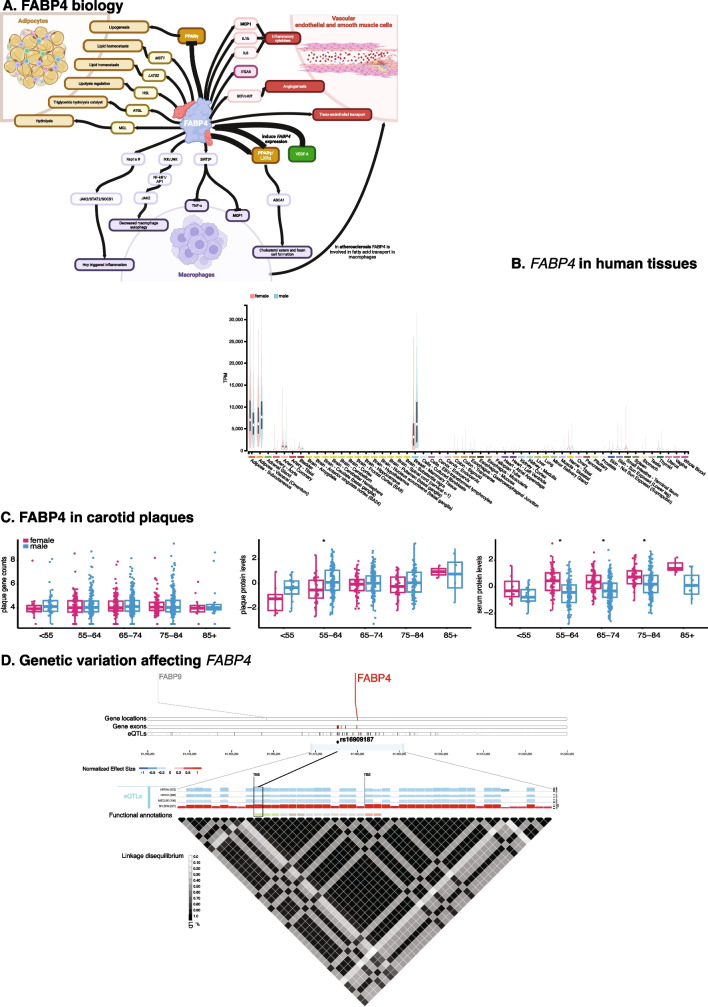
FABP4 in human tissues. **A** Schematic representation of FABP4 interactions in adipocytes, vascular endothelial cells and smooth muscle cells, and macrophages. PPARγ: peroxisome proliferator-activated receptor gamma; MST1: Mammalian sterile 20-like protein 1; LATS2: large tumor suppressors 1; HSL:Hormone sensitive lipase; ATGL: adipose triglyceride lipase; MGL: monoacylglycerol lipase; MCP1: monocyte chemoattractant protein-1; IL-1b: interleukin 1b; IL6: interleukin 6; ITGA5: integrin subunit alpha 5; VEGF: vascular endothelial growth factor; LXRα: liver X receptor alpha; ABCA1: ATP-binding cassette A1; IKK: IκB kinase; JNK: c-Jun N-terminal kinase; NFkB1: nuclear factor kappa B; AP1: activator protein 1; SIRT3: sirtuin 3; TNFα: tumor necrosis factor alpha; Rap1a: Ras-related protein 1a; JAK2: Janus kinase 2; STAT2: signal transducer and activator of transcription 2; SOCS1: suppressor of cytokine signaling 1; Hcy: homocysteine. Tumor necrosis factor α (TNF-α) and monocyte chemoattractant protein-1 (MCP1) are inflammatory cytokines. Bold letters indicate proteins present in more than one cell type. * = in *FABP4-*deficient macrophages; # = Hcy treated [12•, 19]. **B** The *FABP4* gene expression in different human tissues stratified by sex. Data were taken from GTEx Portal (http://www.gtexportal.org/) on 2023–10-10 [113]. *FABP4* is mostly expressed in adipose and mammary tissues and shows low expression in the circulation. **C** *FABP4* gene expression and FABP4 protein levels in carotid plaques (upper and middle panel), and FABP4 protein levels in serum (lower panel) per age stratum and stratified by gender. No differences are observed for *FABP4* expression in carotid plaques per age stratum and gender. Both plaque and serum FABP4 levels rise by age group. However, plaques-derived FABP4 levels are higher in men, whereas serum-derived FABP4 levels are higher in women. Data from the Athero-Express Biobank Study [52, 111, 112], see Methods. **D** Expression quantitative trait loci (eQTLs) for *FABP4* gene expression in human tissues. In this 2000-Kb genomic region on chromosome 8, the *FABP4* gene is marked in red. The 4 bars under the gene names show the gene locations, the *FABP4* gene exons, and the eQTLs for *FABP4*. The middle panel shows the normalized expression effects (NES) for eQTLs per tissue, blue is a negative NES, red is a positive NES. The functional annotations are light red) promotor flanking regions, light grey) intronic variant, dark gray) non-coding transcript exon variant, and green) 3′ UTR variant. HRTAA: atrial appendage in the heart; HRTLV: left ventricle in the heart; MSCLSK: skeletal muscle; SPLEEN: spleen; TES: transcription end site; TSS: transcription start site. The yellow diamond and line demarcate rs16909187 at chr_81478482 (b38) with non-effect allele G and effect allele A. The A-allele is associated with lower *FABP4* gene expression HRTAA, HRTLV, and MSCLSK, whereas the same allele is associated with higher *FABP4* expression in SPLEEN and higher serum FABP4 levels [77••]. The bottom panel shows all the variants, i.e., eQTLs, affecting *FABP4* gene expression and the linkage disequilibrium plot.

One of the earliest mentions of FABP4 in the context of atherosclerosis dates back to 2002, where FABP4, formerly known as ‘adipocyte lipid binding protein’ (ALBP), ‘adipocyte FABP’ (AFABP), or ‘adipocyte P2’ (aP2), was discovered through a subtractive cDNA library screening in oxidized LDL (oxLDL)-treated and control THP-1 macrophages [14]. As an intracellular, high-affinity, selective lipid-binding protein, FABP4 plays a pivotal role in lipolysis, storage of lipids, and facilitates the transport of fatty acids as a chaperone (Fig. 1A) [15, 16]. It is predominantly expressed in white and brown adipocytes, monocytes and macrophages, and endothelial cells[17] and present in both the nucleus and cytoplasm (Fig. 1B). FABP4 is able to differentially bind ligands, and depending on the type of ligand, a conformational change exposes the nuclear localization signal [18]. Activating ligands, such as linoleic acid and troglitazone, lead to exposure and enable the transport of these ligands from the cytosol to the peroxisome proliferator-activated receptor gamma (PPARγ) in the nucleus, a protein associated with adipocyte differentiation, insulin sensitivity, and macrophage function, enhancing its transcriptional activity [18].

Modern-day high-fat diet (HFD)-induced obesity results in expansion of adipose tissue rich in inflammation inducing adipocytes and macrophages and concomitantly increase adipocyte secretion of FABP4 [12•]. Cellular and circulating FABP4 exert different roles in adipocytes, vascular endothelial cells (VECs), VSMCs, and macrophages under healthy and disease conditions. 

### FABP4 in Adipocytes

A fascinating dynamic exists between *PPARG* and *FABP4*. The expression of *FABP4* is highly induced during adipocyte differentiation and under transcriptional control of PPARG agonists, fatty acids, insulin, but also cortisone-like medicine such as dexamethasone [19]. On the other hand, FABP4 plays a role in the regulation of enzyme activity by inducing *PPARG* transcription, while it also triggers PPARγ ubiquitination and subsequent proteasomal degradation [19]. While FABP4 supports the accumulation of surplus fatty acids within lipid droplets in adipocytes, it also possesses a binding site for hormone-sensitive lipase (HSL), a key regulator of lipolysis in adipocytes. While FABP4 lacks the conventional N-terminal signal sequence needed for the classical ER-Golgi-dependent secretion, it is secreted from adipocytes through a series of intracellular triglyceride hydrolysis mediated by adipose triglyceride lipase (ATGL), HSL, and monoacylglycerol lipase (MGL) [19]. In the pursuit of investigating perspective remedies for obesity, human adipose-derived mesenchymal stem cells (hADSCs) were treated with a Boron derivative in conjunction with a pluronic, utilized as a carrier in therapeutic formulations [20]. This treatment resulted in the diminished expression of *PPARG* and *FABP4*, as well as a reduction in the intracellular accumulation of lipid-droplet accumulation within hADSCs. Additionally, the two major regulators, *LATS2* and *MST1*, responsible for governing lipid homeostasis in the context of adipocyte differentiation and proliferation, exhibited decreased expression [20]. Post-translational modification, such as phosphorylation (also see ‘FABP4 and the macrophage’), can significantly alter its properties and influences the interactions with other molecules [21]. To explore how modifications may contribute to obesity-induced insulin resistance and type 2 diabetes, the acetyl-lysine proteome profile (acetylome) was studied in subcutaneous and omental adipose tissue [22]. In insulin-resistant subcutaneous fat, FABP4 protein levels were highest, accompanied by a notable increase in acetylation [22]. During adipocyte differentiation, FABP4 protein exhibited a distinctive localization pattern: primarily within the nucleus at early stages, and shifting to the cytosol in later stages [22]. Introducing site-directed mutations targeting acetyl-sites in differentiating adipocytes resulted in substantial alterations in the distribution of FABP4 between the nucleus and the cytosol, reduced secretion of FABP4, changes in intracellular lipid-droplet accumulation, and increased levels of HSL in the mutants compared to the wildtype cells [22].

### Effects of FABP4 on Vascular Endothelial and Smooth Muscle Cells

Under normal physiological conditions, *FABP4* is expressed in vascular endothelial cells of the capillaries and small veins, but not the arteries. It is involved in trans-endothelial transport of fatty acids, regulated by PPARγ, to fatty acid consuming tissues, such as the heart, skeletal muscle, and kidneys [19, 23]. In vascular endothelial cells, vascular endothelial growth factor A (*VEGFA*) is a potent regulator of *FABP4* which in turn upregulates intracellular signaling pathways, while down-regulation of *FABP4* reduces cellular proliferation and angiogenesis in response to *VEGFA* [24]. FABP4 contributes to endothelial cell proliferation and migration, and through the modulation of the stem cell factor/c-kit pathway acts as a pro-angiogenic agent [24]. Endothelial cell derived FABP4 is also shown to increase pro-inflammatory cytokines, and proliferation and adhesion-related proteins, such as MCP1, IL1b, IL6, and ITGA5, inducing vascular smooth muscle cell proliferation and migration [25]. In patients with coronary artery disease (CAD), there is a remarkable interaction between human coronary arterial cells (HCAECs) and mononuclear cells (MNCs) [26]. The supernatant from MNCs isolated from CAD patients exhibited elevated levels of FABP4, and HCAECs from CAD patients displayed increased adherence to MNCs compared to controls [26]. This effect could be mitigated by the use of a FABP4-antibody [26]. The enhanced cellular adhesion was induced by oxidized-LDL (oxLDL), a modified form of lipids and apolipoprotein B resulting from lipid peroxidation, and a well-known atherogenic agent contributing to the formation of macrophage foam cells [27]. Treatment with oxLDL leads to higher levels of FABP4 proteins and adhesion molecules, including ITGB2, ITGA4, and PSGL1, in the supernatant of CAD patients [26]. However, the use of a FABP4-antibody reduced the expression of these integrins [26].

### FABP4 and the Macrophage

It has been known for some time that FABP4 inhibits PPARγ/LXRα and its downstream ABCA1 pathway, which causes the formation of cholesterol esters and foam cells in macrophages [28, 29]. Suppression of FABP4 reduces PPARγ activity, and consequently, foam cell formation by M2 macrophages is impaired [30]. The expression of inflammatory cytokines, including TNF-α and MCP1, is reduced in *FABP4*-deficient macrophages, and this effect is mediated through SIRT3 [31]. In co-culture experiments of (murine) macrophages and adipocytes, the production of inflammatory cytokines is significantly reduced in the presence of butyrate (which inhibits adipocyte lipolysis), possibly through a pathway involving p38, ERK1/2, JNK1/2, and IκB/NF-κB [32, 33]. FABP4 also activates the IKK/JNK pathway, which leads to inflammatory responses by NF-κB/AP1 pathways, and attenuates Janus kinase 2 (JAK2) activity, which leads to a decrease in macrophage autophagy [34]. Hypomethylation of *FABP4* accelerates lipid accumulation in homocysteine-induced (Hcy) atherosclerosis, implying that FABP4 may be involved with foam cell formation and macrophage inflammation [21, 35]. Apolipoprotein E knockout *(ApoE*^−/−^) mice treated with Hcy show acceleration of atherosclerosis and activated macrophage inflammation [21]. FABP4 plays a role in promoting macrophage inflammation triggered by Hcy by activating the Janus kinase 2/signal transducer and activator of transcription 2 (JAK2/STAT2) pathway. Ras-related protein (Rap1a) acts as an intermediary in the FABP4-mediated regulation of this pathway during macrophage inflammation.

Moreover, the post-translational phosphorylation of c-Src at Tyr416 appears to be essential for the activation of the pathway. Interestingly, the p-Tyr416 level of c-Src and its membrane localization significantly increased with FABP4, while the knockdown of suppressor of cytokine signaling 1 (*SOCS1*) led to increased secretion of proinflammatory factors in *FABP4*-overexpressing macrophages, an effect that was reversed by Rap1a knockdown [21]. It is worth noting that polybrominated diphenyl ethers (PBDEs), which are commonly utilized as flame retardant in numerous industrial and consumer products, possess obesogenic properties and have the ability to bind to PPARγ, thus triggering adipocyte differentiation [36]. PBDEs can also activate PPARγ in THP-1 macrophages, leading to an increase of expression of *FABP4* and *CD36*. CD36, functioning as a scavenger receptor mediating the uptake of free fatty acids and oxLDL in macrophages[37], is upregulated upon PPARγ transactivation through PBDE. This, in turn, promotes the uptake of oxLDL and the accumulation of lipids, ultimately resulting in the formation of foam cells [36].

### FABP4 in Animal Studies

Animal studies have clearly shown that FABP4 is associated with atherosclerotic plaque formation and vulnerable plaque features. Mice lacking both *ApoE* and *Fabp4* develop markedly smaller atherosclerotic lesions [38] and contained fewer macrophages than wildtype *ApoE-/-* mice. In addition, the lack of *Fabp4* in donor marrow cells leads to the development of smaller atherosclerotic lesions in the recipient mice, demonstrating the potential role of macrophage expressed * Fabp4* in lesion development [39]. Importantly, these studies show that the onset of the metabolic syndrome occurs via expression of Fabp4 in adipocytes, while the initiation of atherosclerotic plaques occurs through Fabp4 expression in macrophages [38, 40, 41]. The *Fabp4-*deficient macrophages showed alterations in inflammatory cytokine production and a reduced ability to accumulate cholesterol esters when exposed to modified lipoproteins [40]. A drug intervention study in atherosclerotic mice demonstrated that an orally active small-molecule inhibitor of FABP4, BMS309403 (see also “Known drugs and compounds inhibiting FABP4”), was an effective therapeutic agent against severe atherosclerosis and type 2 diabetes in mouse models [42].

Metabolic syndrome describes a multifactorial process of multiple interrelated cardiovascular risk factors, including obesity, hyperlipidemia, and insulin resistance, and increased risk of cardiovascular disease [43]. Indeed, overexpression of a specific microRNA (miR-100) resulted in a reduced weight gain in high-fat fed mice compared to wildtype mice (on the same diet) [44]. These miRNA-100 overexpressing mice show marked decreased expression  *Cd36*, *Pparg*, and *Fabp4 *in hepatocytes, reducing hepatic lipid accumulation and inflammation, while demonstrating increased energy expenditure [44]. Thus, hepatic Fabp4 may be an important factor in high-fat diet induced metabolic syndrome and subsequent atherosclerotic disease [44]. Not only selective drugs but also specific nutritional compounds may be able to reduce metabolic syndrome, and in C57BL/6 J mice on a high-fat diet Akebia saponin D, found in plants used in herbal tea, was shown to have beneficial effects on the microbiome [43]. This compound was shown to reduce lipid-induced tight-junction damage in intestinal epithelial cells through the Pparg/Fabp4 pathway, while the use of a PPARγ-inhibitor partially blocked these beneficial effects [43].

Similar to humans (Fig. 1C), FABP4 rises as mice age [45]. Intuitively, FABP4 is also positively correlated with LDL-rich cholesterol and triglycerides, but negatively with HDL-rich cholesterol [45]. When *Fabp4* is silenced in these aging mice, they show increased metabolic activity, while at the same time fasting blood glucose and insulin decrease, and reduced hepatic lipid deposition [45]. Hepatic transcriptomic analyses in *Fabp4*-silenced aging mice reveals a transcriptomic reprogramming evident from reduced oxidative phosphorylation and increased TGF-β signaling [45], while overexpression of Fabp4 induces cellular senescence [45].

## Observational Studies of FABP4

### A Long History

The association of circulating FABP4 levels with cardiovascular disease has been studied extensively in observational studies. Results from a Japanese population-based study show, that compared to other FABPs, FABP4 levels are highest in the general population, and between the sexes, females have higher FABP4 levels attributed to the higher ratio of body fat in women than men [46]. FABP4 is also associated with known cardiovascular risk factors including body mass index (BMI), blood pressure, high-sensitive CRP, cholesterols, carotid intima-media thickness (cIMT), and negatively correlated to estimated glomerular filtration rate (eGFR) [19, 46, 47]. The latter suggesting that FABP4 is mainly excreted from the body through the renal clearance [19, 46]. Indeed, in the same general population from Japan, urinary FABP4 is correlated to albuminuria and associated with yearly decline in eGFR in the Tanno-Sobetsu Study [48].

In a Chinese population-based study individuals without cardiovascular disease were followed for a median duration of 9.4 years and 182 out of 1847 included developed CVD [49]. Circulating levels of FABP4 were highest in individuals with CVD, and Cox-proportional hazard analyses show FABP4 was associated with incident CVD over a period of 12 years. In another study involving the same cohort FABP4 was shown to correlate with glucose dysregulation, and was predictive for the development of type 2 diabetes in individuals without a previous history of diabetes [50]. In a German study, 1069 patients with previous coronary artery disease were followed for a duration of 10 years and circulating FABP4 levels increased as the number of metabolic syndrome components increased, and more so in females compared to males [51]. Cox regression analyses in the same study show that high FABP4 levels are associated with risk of secondary CVD during follow-up. This aligns with results from two other studies including patients undergoing carotid endarterectomy (CEA), where plaque-derived FABP4 levels were shown to be higher in symptomatic patients than asymptomatic patients [52, 53], and FABP4 gene expression was higher in carotid plaques from symptomatic patients within 1-month preceding CEA [53]. High plaque levels of FABP4 are also associated with a higher risk of secondary cardiovascular events in the three years post-event [52]. In addition, in patients with end-stage renal disease, as well as in patients with type 2 diabetes, FABP4 levels are predictive of cardiovascular death [54, 55]. A similar study in 1316 patients with chronic kidney disease also showed that FABP4 levels, ascertained using the multiplex proximity extension assay (PEA) technique [56], were associated with secondary major cardiovascular events (MACE); however, this could not be replicated in a cohort of 300 patients [57].

### FABP4 as a Marker of Disease in At-Risk Populations

These studies are supported by more recent work. In a prospective, population-based cohort study including 5888 individuals aged 65 years and over, circulating FABP4 levels were higher in women than in men[58]. While FABP4 was not associated with incident myocardial infarction or stroke, it was associated with incident cardiovascular death among 4026 individuals free of CVD during follow-up [58]. In 681 patients with prevalent CVD, FABP4 was associated with cardiovascular death even after correction for confounders [58]. In a South African study involving black and white participants FABP4 was measured using the multiplex PEA technique and correlated to several phenotypes of vascular health [59]. FABP4 was associated with cIMT and pulse wave velocity, a marker of arterial stiffness and an independent predictor of cardiovascular risk, but not hypertension [59]. A recent update of the aforementioned Japanese Tanno-Sobetsu Study again confirmed that FABP4 levels are higher in women than men in the general population, and following individuals prospectively for 12 years shows FABP4 levels are predictive of cardiovascular death after adjustment for cardiovascular risk factors [60].

Conversely, a large study involving over 27,000 participants shows that FABP4 is associated with risk of type 2 diabetes, and potentially stroke risk, but not with myocardial infarction [61]. Other data also suggest FABP4 was higher in type 2 diabetes patients compared to healthy individuals [62], and may present a biomarker for cardiovascular disease given its association with arterial stiffness, renal function, adiposity, and hypertriglyceridemia [63, 64]. In high-fat diet fed mice, FABP4 was elevated in plasma and cardiac cells showed higher triglyceride loads [62]. The latter was further investigated in vitro where cardiac cells were challenged with extracellular FABP4 and showed high intracellular lipid content, which could be blocked through inhibition with a FABP4-specific drug [62].

Other studies show association of FABP4 levels with elongated QT intervals in patients with stable angina and kidney disease [65], but also with rapid renal functional decline in patients with diabetes [66]. A study of 973 patients undergoing coronary intervention shows FABP4 associated with cardiovascular outcome [67]. In type 2 diabetes patients, FABP4 levels were positively associated with peripheral artery disease [68], similar to a study involving hemodialysis patients [69]. A study in 777 Europeans applied the same PEA technique to measure FABP4 and found a strong correlation with leptin, a marker of the amount of adipose tissue and the development of CVD, both in diabetic patients and the general population [70], and especially in men. Longitudinal analyses showed that the association of leptin with risk of MACE was slightly attenuated by adding FABP4 to the model, suggesting a potential mediation effect [70].

## Genetics of FABP4 in Humans

The FABP4 gene, encoding the FABP4 protein, is located on chromosome 8q.21.13 and evolutionarily conserved with a four-exon organization and 4 known isoforms. Several candidate gene studies reported on associations of arbitrarily chosen common DNA sequence variations in or near *FABP4* with its expression or FABP4 protein levels across differing tissues and ancestral diverse populations. One common variant (rs77878271, T-87C), discovered through resequencing in 98 individuals [71], is located in the promoter region of *FABP4*. Genotyping of 7900 individuals from two large prospective population studies associated this variant with significantly lower triglycerides levels, lower risk of coronary heart disease, and diabetes [71]. The same variant was also reported to associate with lower prevalence of carotid plaque, reduced carotid intima-media thickness and total cholesterol levels, and lower odds of myocardial infarction [72]. Conversely, in a large population study, this variant was not associated with diabetes risk in ancestry-specific and pooled analyses [73]. Other variants, most in linkage disequilibrium to some extent, were also associated with FABP4 levels and fasting glucose, but not insulin, in the circulation of older individuals. More recently, this variant was shown to decrease *FABP4* expression in epicardial adipose tissue [74] and associates with risk for cardiovascular disease in type 1 diabetes patients.

### Large-Scale Genetics Discoveries

Most of these studies predate, or were on the cusp of, the era of genome-wide association studies and lacked robust study designs or methods, potentially leading to under-powered studies and irreproducible associations. Only a few GWAS were executed and reported on agnostically discovered common variants associated with FABP4 circulating levels [75, 76]. Of course, biologically, it makes sense that common variants near or in any given gene, its transcription start site, or its enhancer regions, should modulate expression in the context of the Central Dogma where DNA encodes RNA that is translated to protein. The large-scale Genotype-Tissue Expression (GTEx) Project includes over 50 different tissues in which gene expression was measured through RNA sequencing and the samples were genotyped (Fig. 1D). Analyses within this dataset robustly show that *FABP4* is expressed in most tissues, showing few sex-specific differences, and highest expression in adipose and mammary tissues. Confirming earlier reports, *FABP4* is expressed in atherosclerotic plaques, but no apparent sex differences can be seen, whereas FABP4 protein levels do differ between the sexes in both plaque and serum. Many common variants in and near *FABP4* modulate its expression in some, but not all, tissues, specifically in heart tissues, skeletal muscle, and spleen (Fig. 1C, D). Most recently, a large-scale meta-analysis including over 30,000 individuals looked at the genetics of circulating levels of 90 proteins, including FABP4 [77••]. Mainly focused on the discovery of druggable targets, it did discover two loci, one near *FABP4* and one near *PPARG*, showing the strongest association with FABP4 levels in the blood [77••]. Using a different methodology to measure FABP4, the INTERVAL study showed a positive and causal correlation of FABP4 with BMI in 2737 healthy participants [78]. Another study provided evidence of a large polygenic component to FABP4 protein levels, and genetic correlation analyses shows that variants affecting FABP4 protein levels are also positively correlated with coronary artery disease, type 2 diabetes, waist-hip-ratio, and creatinine [79].

## Known Drugs and Compounds Inhibiting FABP4

There are 12 different chemical classes that are known to have an inhibitory effect on FABPs: pyrazole derivatives, oxazole derivatives, imidazole derivatives, indole derivatives, benzimidazole derivatives, thiophene and thiazole derivatives, pyrimidine, bicyclic pyridine and quinoxaline derivatives, urea and carbamoyl derivatives, sulfonamide derivatives, triazole derivatives, 2-aminobenzoic acid derivatives, and other compounds like natural compounds or FDA-approved drugs [80].

Drugs or compounds known for inhibition of FABP4 affect either the initiation of expression (indirect effect) or block the fatty acid binding site of the protein (direct effect). The best known is BMS309403, a small molecule that competitively inhibits fatty acid binding sites and thus directly has an effect on FABP4 activity [42, 81]. Another compound with similar functionality is HTS01037 [82]. The downside of these compounds is the relative high affinity for FABP3, or heart FABP, which can lead to cardiotoxicity given its high expression in cardiac tissue [19]. Currently, there are no clinical trials involving FABP4 inhibitors due to this side effect, along with metabolic issues and drug resistance [80].

The search for the most optimal compound is ongoing. A class of biphenyl scaffold molecules are shown to have promising inhibitory properties [83••]. To make the most optimal inhibitor with best specificity, compound 10g was developed and with a dose of 25 μM this compound inhibits FABP4 92% and FABP3 26% [84]. The inhibition by BMS309403 is higher for FABP4 (98%), but this is also the case for FABP3 (53%). Although the selectivity of BMS309403 for FABP4 is better, the unintended side effects are considerable due to high selectivity for FABP3, which leads to cardiotoxicity [84]. The inhibitory constant for BMS309403 and 10g are similar for FABP4 (respectively 0.36 μM and 0.51 μM), but for FABP3, 10g is more favorable (respectively, 30 μM and 33 μM) [84]. Other compounds resulting from tweaking this inhibitory protein are 16dk, 16do, and 16du [84].

The expression of FABP4 can be indirectly affected by inhibition of other pathways. Metformin, for example, is a hypoglycemic drug with an effect on macrophage-induced inflammation [85]. It is known to inhibit inflammation, reduce oxidative stress, lower foam cell formation, and induce apoptosis of macrophages in type 2 diabetes patients. The expression of  FABP4. is indirectly reduced via inhibition of FOXO1-induced FABP4 transcription in macrophages by metformin [85].

Most drugs or compounds have been tested and researched in vitro with murine 3T3-L1 adipocytes, human monocytic leukemia THP-1 macrophages, but were also tested in vivo with leptin receptor-deficient and high-fat diet-induced mouse models [30, 43, 86]. There are some studies conducted into the effect of antidiabetic drug (Sitagliptin) or LDL-cholesterol lowering drug (Anagliptin, a DPP-4 inhibitor) on the gene expression or protein serum levels of FABP4 [87]. The latter, anagliptin, showed reduced serum FABP4 levels independent of HbA1c or LDL-cholesterol levels.

By combining machine learning and molecular docking on the approximately 2600 compounds in the FDA-approved drug library (https://go.drugbank.com), four different compounds (cobimetinib, larotrectinib, pantoprazole, and vildagliptin) were identified [88]. Cobimetinib, a mitogen-activated protein kinase (MEK)-specific small molecule inhibitor, is a drug used in melanoma (cancer) research and out of the four the most effective inhibitor of FABP4 [89, 90]. The compound is thought to inhibit the phosphorylation of JNK/c-Jun by FABP4, which might lead to a decrease in inflammatory response factors.

## Future Perspective on FABP4 Research

Clearly considering FABP4 as an adipocyte-only-FABP is oversimplifying matters. Preclinical work has shown that FABP4 is critical for intracellular fatty acid transportation in the endothelium, affecting cellular adherence and altering cytokine expression [25]; stimulation through VEGFA and PPARγ induces endothelial cell proliferation and angiogenesis [91]. Ectopic endothelial expression of FABP4 induces an inflammatory response from VSMCs and increase proliferation and migration [25]. In macrophages, FABP4 is critical to the inflammatory response, oxLDL uptake, intracellular lipid accumulation, and concomitant foam cell formation [21]. In both mice and men, *FABP4* expression and protein levels, in lipid-rich macrophages in early and advanced atherosclerotic plaques, is elevated [52, 92–94]. Results from observational studies convincingly position serum FABP4 as a biomarker for cardiovascular disease, specifically in at-risk populations with type 2 diabetes or kidney disease. In point of fact, a potent FABP4 inhibitor was shown to reduce atherosclerosis and reduces inflammation while increasing insulin sensitivity of adipose tissues in obese and diabetic mice [42].

Yet, although literature on FABP4 points to a clear predictive value of elevated serum FABP4 for atherosclerotic disease and cardiovascular death [60], clinical interventional studies inhibiting protein expression are lacking. This is surprising because studies of human atherosclerotic plaques show that FABP4 can be recognized as a marker that is strongly associated with vulnerable plaque characteristics. High local plaque FABP4 expression levels are strongly associated with an inflammatory unstable plaque composition and with symptomatic lesions [52]. In addition to these cross-sectional observations in human samples, plaque FABP4 protein levels are strongly predictive for systemic cardiovascular outcome during 3-year follow-up [52]. Still, despite these results being promising, FABP4 inhibitors are not available for clinical use, which may be associated with unsatisfactory efficacy and physicochemical properties [83••].There are a few compounds, some of which are approved drugs for type 2 diabetes, that directly affect FABP4, or its downstream pathways. Future studies should focus on the adverse and therapeutic effects of these drugs and specifically address effects on FABP4 in the context of atherosclerosis. Additionally, as post-translational modification appears to be critical for the properties and the cellular localization of FABP4, the development of site-directed compounds, that for instance alter acetylation or phosphorylation, could be of interest.

Another factor in play, is the central role FABP4 has in lipid homeostasis in both health and at-risk individuals with obesity, decreased kidney function, and/or type 2 diabetes. Large-scale genetic studies have robustly associated common variants near *FABP4* and *PPARγ* with FABP4 serum levels. Interestingly, the same alleles that affect FABP4 in serum one direction may affect its gene expression differently in other tissues (Fig. 1), but how these affect expression in macrophages in the vessel is unknown. Comprehensive molecular QTL analyses to investigate how genetic variation impacts protein levels and gene expression in both adipocytes and vascular cells are needed, while being considerate on the different measurement platforms used [95]. These studies should encompass samples from both healthy individuals and those with evident atherosclerosis to unveil genetic effects specific to the disease.

Because of the invariant nature of the human genome and the random distribution of alleles from parents to offspring at conception, the effects of genetic variation should be free of confounding by traditional risk factors and not influenced by disease status (reverse causality) [96]. In essence, we are all part of a natural clinical trial, in which genetic risk is randomly distributed and unaffected by confounding (Mendelian randomization (MR)). MR studies are a powerful means to gauge the causal relation between a putative biomarker (and implicit druggable target) and disease [97–99]. However, both observational studies and genetic correlation analyses show profound positive correlations of FABP4 serum levels and genetic variation with markers of cardiometabolic health, including BMI, kidney function, serum lipid levels, and type 2 diabetes. This makes inference of whether FABP4 is causal to CVD challenging as collider bias, where FABP4 (exposure) and cardiovascular disease (outcome) are both influenced by other factors, may muddy the water [100, 101]. Additional research should focus on specific at-risk subgroups and apply strategies to address collider bias by other factors—in this case kidney function, lipid levels, BMI, and type 2 diabetes.

Prenatal malnutrition can have a profound impact on health in the human offspring [102]. DNA methylation acts as a mediator between transient adverse environmental factors in early life and metabolic health risk in adulthood [103]. In humans, FABP4 has been shown to vary during lactation and shows strong correlations with adiponectin and leptin in breast milk [104], and maternal adiposity and breast milk composition may be linked to infant growth [105]. At the same time, weight trajectories in individuals with different polygenic risks for obesity start to diverge in early life [106], and the effects of an obesogenic environment on health are most pronounced in those with a genetic predisposition [106, 107].

It is intriguing to hypothesize how FABP4 may be epigenetically influenced by maternal adiposity and breast milk composition and to study what the mediating effects of FABP4 may be in those genetically predisposed for obesity and concomitant poorer cardiovascular health. Likewise, other environmental factors, including smoking, are known to affect DNA methylation of the transcriptome in atherosclerosis [108]. As technology evolved, we are now able to partially disassociate chronological age from biological age and show how biological age may result in mesenchymal reprogramming [109]. It will be intriguing to further dissect the molecular mechanisms through which natural or man-made environmental factors change the FABP4-associated methylome and thereby influence atherogenesis. Specifically, it would be of interest to investigate whether FABP4 is involved in cellular phenotype switching through reprogramming of macrophages and vascular endothelial and smooth muscle cells.

Curing disease and prolonging life has been a grand human endeavor since the age of Aristotle. Whether FABP4 will be a means to an end will hopefully crystallize in the next decade.

## Methods

### Literature Search

We used PubMed to collect the cumulative knowledge on FABP4 in literature, 6619 articles in Dutch or English involved the keyword “FABP4” (October 2023) in animals, including humans. We narrowed the search parameters based on the query below and found 356 articles on FABP4 in atherosclerotic disease. These were supplemented with 4 additional articles covering a genetic study of circulating protein levels [77••, 79, 110]. We used this set as the starting point for this review:

(“fabp4” OR “aP2” OR “A-FABP” OR “AFABP” OR “ALBP”) and (“cardiovascular disease” OR “coronary artery disease” OR atherosclerosis OR “atherosclerotic disease” OR “atherosclerotic plaque”)

We downloaded this dataset as a.nbib file from PubMed and collected the abstracts and checked accessibility through PaperPile (www.paperpile.com).

### Statistical Analyses, Data, and Code Availability

We used data from the Athero-Express Biobank Study, an ongoing study including patients undergoing carotid endarterectomy in The Netherlands [111]. Routinely plaque material with associated clinical data and blood samples are collected. The local medical ethics committee approved of the study, all patients provided written consent, and the study adheres to the guidelines of the Helsinki Declaration.

For the purposes of this review, we compared the expression of *FABP4* in plaque [112], and the FABP4 protein levels in plaques and the circulation [52]. We previously described the collection of carotid plaques for the transcriptomic measurements, and after quality control, we normalized and log-transformed expression [112]. The protein levels were normalized by subtracting the mean from each value and divided this by the standard deviation. To test for significant differences between groups, we applied Kruskal–Wallis tests. All data are available through DataverseNL (doi.org/10.34894/4IKE3T) and codes are available through GitHub (github.com/CirculatoryHealth/Review_FABP4).
